# 2-Chloro-4-(1*H*-pyrazol-1-yl)-5-(trifluoro­meth­yl)pyrimidine

**DOI:** 10.1107/S1600536810018854

**Published:** 2010-05-26

**Authors:** Kevin D. Bunker, Curtis Moore, Cynthia L. Palmer, Arnold L. Rheingold, Alex Yanovsky

**Affiliations:** a Pfizer Global Research and Development, La Jolla Labs, 10770 Science Center Drive, San Diego, CA 92121, USA; b Department of Chemistry and Biochemistry, University of California, San Diego, 9500 Gilman Drive, La Jolla, CA 92093, USA

## Abstract

The reaction of 2,4-dichloro-5-(trifluoro­meth­yl)pyrimidine with 1*H*-pyrazole gave two structural isomers in a 1:1 ratio that were separable by chromatography. The title compound, C_8_H_4_ClF_3_N_4_, was the first product to elute and was characterized in the present study to confirm that substitution by the pyrazolyl group had occurred at position 4. The mol­ecule (with the exception of the F atoms) is essentially planar, with a mean deviation of 0.034 Å from the least-squares plane through all non-H and non-F atoms. The bond angles in the pyrimidine ring show a pronounced alternating pattern with three angles, including those at the two N atoms being narrower, and the remaining three wider than 120°.

## Related literature

For the structures of similar pyrazolylpyrimidine derivatives, see: Peresypkina *et al.* (2005 ▶); Liu *et al.* (2005 ▶); Brunet *et al.* (2007 ▶). For statistics on endocyclic angular distortions in triazine derivatives similar to those observed in the title compound, see: Allington *et al.* (2001 ▶).
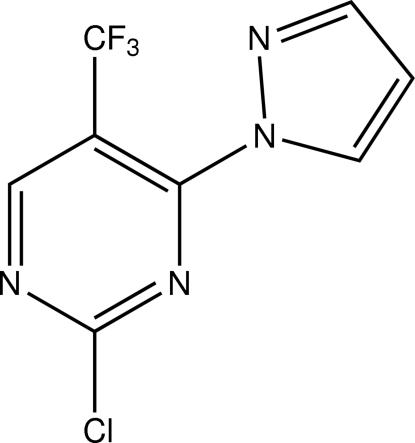


## Experimental

### 

#### Crystal data

C_8_H_4_ClF_3_N_4_
*M* *_r_* = 248.60
Orthorhombic, 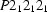

*a* = 5.5776 (3) Å
*b* = 7.7117 (4) Å
*c* = 21.8335 (12) Å
*V* = 939.12 (9) Å^3^
*Z* = 4
Cu *K*α radiation
μ = 3.90 mm^−1^
*T* = 100 K
0.40 × 0.21 × 0.10 mm

#### Data collection

Bruker APEXII CCD diffractometer
Absorption correction: multi-scan (*SADABS*; Bruker, 2001 ▶) *T*_min_ = 0.305, *T*_max_ = 0.697
3416 measured reflections
1402 independent reflections
1273 reflections with *I* > 2σ(*I*)
*R*_int_ = 0.030
θ_max_ = 61.9°

#### Refinement

*R*[*F* ^2^ > 2σ(*F* ^2^)] = 0.033
*wR*(*F* ^2^) = 0.082
*S* = 1.02
1402 reflections
145 parameters
H-atom parameters constrained
Δρ_max_ = 0.21 e Å^−3^
Δρ_min_ = −0.22 e Å^−3^
Absolute structure: Flack (1983 ▶), 503 Friedel pairs
Flack parameter: 0.05 (2)

### 

Data collection: *APEX2* (Bruker, 2007 ▶); cell refinement: *SAINT* (Bruker, 2007 ▶); data reduction: *SAINT*; program(s) used to solve structure: *SHELXS97* (Sheldrick, 2008 ▶); program(s) used to refine structure: *SHELXL97* (Sheldrick, 2008 ▶); molecular graphics: *SHELXTL* (Sheldrick, 2008 ▶); software used to prepare material for publication: *SHELXTL*.

## Supplementary Material

Crystal structure: contains datablocks global, I. DOI: 10.1107/S1600536810018854/ez2213sup1.cif

Structure factors: contains datablocks I. DOI: 10.1107/S1600536810018854/ez2213Isup2.hkl

Additional supplementary materials:  crystallographic information; 3D view; checkCIF report

## Figures and Tables

**Fig. 1.**
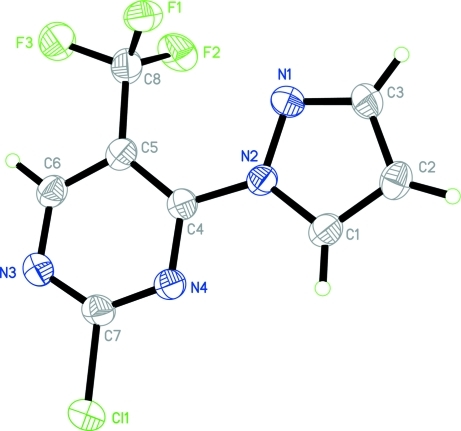
Molecular structure of the title compound, showing 50% probability displacement ellipsoids and atom numbering scheme. H atoms are drawn as circles with arbitrary small radius.

## supplementary crystallographic information

### Comment

The reaction of 2,4-dichloro-5-(trifluoromethyl)pyrimidine with 1*H*-pyrazole gave two structural isomers in a 1:1 ratio that were separable by chromatography. The title compound was the first product to elute and was characterized in the present study to confirm substitution by N-pyrazolyl group to have occurred at position 4 (Fig. 1).

The molecule (with the exception of the F atoms) is essentially planar: the maximum displacement of the N1 atom from the plane, drawn through all non-F and non-H atoms, is equal to 0.076 (4) Å. Other pyrazolylpyrimidine derivatives were also shown to have planar molecules (Peresypkina *et al.*, 2005; Liu *et al.*, 2005; Brunet *et al.*, 2007).

The geometry of the pyrimidine ring is characterized by alternating of bond angle distortions: angles at the N3, N4 and C5 atoms [112.8 (3); 116.1 (3); 115.4 (3)°] are all narrower, whereas the remaining angles in the ring at the C4, C6 and C7 atoms [121.2 (3); 124.7 (3); 129.7 (3)°] are wider than 120°. Such angular distortions were also observed in other pyrimidine structures (see, for instance, the above quoted papers). The study by Allington *et al.* (2001) contains analysis of some statistics on similar angular distortions in the triazine derivatives.

The dramatic difference between the exocyclic bond angles C4—C5—C8 127.1 (3)° and C6—C5—C8 117.5 (3)° can be attributed to the repulsion of the CF_3_-group from pyrazolyl substituent (the F1···N1 and F2···N1 distances are 2.720 (3) Å and 2.774 (3) Å respectively).

### Experimental

2-Chloro-4-(1*H*-pyrazol-1-yl)-5-(trifluoromethyl)pyrimidine and 4-chloro-2-(1*H*-pyrazol-1-yl)-5-(trifluoromethyl)pyrimidine. To a *N*,*N*-dimethylacetamide (46.0 ml) solution of pyrazole (726 mg, 10.7 mmol) and potassium carbonate (3.84 g, 27.8 mmol) was added 2,4-dichloro-5-trifluoromethyl-pyrimidine (2.01 g, 1.250 ml, 9.27 mmol) by syringe in one shot at rt. The mixture was stirred overnight and monitored by TLC (20% EtOAc/heptane). After consumption of starting material, the reaction mixture was diluted with water and extracted with EtOAc (3×). The organic layers were combined, dried, and concentrated. The crude residue was subjected to flash chromatography (silica gel, 0-40% EtOAc/heptane), and three major bands eluted. The first major band was isolated to give 460 mg (20%) of 2-chloro-4-(1*H*-pyrazol-1-yl)-5-(trifluoromethyl)pyrimidine. The second band was also collected to give 460 mg (20%) of 4-chloro-2-(1*H*-pyrazol-1-yl)-5-(trifluoromethyl)pyrimidine. The third band was found to be 2,4-di(1*H*-pyrazol-1-yl)-5-(trifluoromethyl)pyrimidine and was not isolated. X-ray quality crystals of the first product to elute were grown in DCM/heptane upon slow evaporation.

2-chloro-4-(1*H*-pyrazol-1-yl)-5-(trifluoromethyl)pyrimidine: ^1^H NMR (400 MHz, CHLOROFORM-*d*) δ ppm 6.56 (dd, J=2.77, 1.51 Hz, 1 H) 7.89 (d, J=0.76 Hz, 1 H) 8.59 (dd, J=2.77, 0.76 Hz, 1 H) 8.96 (s, 1 H). ^13^C NMR (101 MHz, CHLOROFORM-*d*) δ ppm 109.96 (s, 1 C) 111.39 - 112.97 (m, 1 C) 117.69 - 126.63 (m, 1 C) 130.42 (s, 1 C) 145.57 (s, 1 C) 155.57 (s, 1 C) 160.86 (q, J=7.09 Hz, 1 C) 163.04 (s, 1 *C*).

4-chloro-2-(1*H*-pyrazol-1-yl)-5-(trifluoromethyl)pyrimidine: ^1^H NMR (400 MHz, CHLOROFORM-*d*) δ ppm 6.57 (dd, J=2.77, 1.51 Hz, 1 H) 7.90 (d, J=1.01 Hz, 1 H) 8.59 (dd, J=2.77, 0.50 Hz, 1 H) 8.92 (s, 1 H). ^13^C NMR (101 MHz, CHLOROFORM-*d*) δ ppm 110.22 (s, 1 C) 121.68 (q, J=272.65 Hz, 1 C) 120.34 (q, J=33.99 Hz, 1 C) 130.23 (s, 1 C) 145.59 (s, 1 C) 156.64 (s, 1 C) 158.07 (q, J=5.14 Hz, 1 C) 161.02 (s, 1 C).

### Refinement

All H atoms were placed in geometrically calculated positions (C—H 0.93 Å) and included in the refinement in riding motion approximation. The *U*_iso_(H) were set to 1.2*U*_eq_ of the carrying atom.

### Crystal data

**Table:**

C_8_H_4_ClF_3_N_4_	*F*(000) = 496
*M**_r_* = 248.60	*D*_x_ = 1.758 Mg m^−^^3^
Orthorhombic, *P*2_1_2_1_2_1_	Cu *K*α radiation, λ = 1.54178 Å
Hall symbol: P 2ac 2ab	Cell parameters from 2204 reflections
*a* = 5.5776 (3) Å	θ = 4.1–61.5°
*b* = 7.7117 (4) Å	µ = 3.90 mm^−^^1^
*c* = 21.8335 (12) Å	*T* = 100 K
*V* = 939.12 (9) Å^3^	Rod, colourless
*Z* = 4	0.40 × 0.21 × 0.10 mm

### Data collection

**Table:**

Bruker APEXII CCD diffractometer	1402 independent reflections
Radiation source: fine-focus sealed tube	1273 reflections with *I* > 2σ(*I*)
graphite	*R*_int_ = 0.030
φ and ω scans	θ_max_ = 61.9°, θ_min_ = 4.0°
Absorption correction: multi-scan (*SADABS*; Bruker, 2001)	*h* = −6→6
*T*_min_ = 0.305, *T*_max_ = 0.697	*k* = −8→8
3416 measured reflections	*l* = −25→24

### Refinement

**Table:**

Refinement on *F*^2^	Secondary atom site location: difference Fourier map
Least-squares matrix: full	Hydrogen site location: inferred from neighbouring sites
*R*[*F*^2^ > 2σ(*F*^2^)] = 0.033	H-atom parameters constrained
*wR*(*F*^2^) = 0.082	*w* = 1/[σ^2^(*F*_o_^2^) + (0.048*P*)^2^] where *P* = (*F*_o_^2^ + 2*F*_c_^2^)/3
*S* = 1.02	(Δ/σ)_max_ = 0.001
1402 reflections	Δρ_max_ = 0.21 e Å^−^^3^
145 parameters	Δρ_min_ = −0.22 e Å^−^^3^
0 restraints	Absolute structure: Flack (1983), 503 Friedel pairs
Primary atom site location: structure-invariant direct methods	Flack parameter: 0.05 (2)

### Special details

**Table:**

Geometry. All esds (except the esd in the dihedral angle between two l.s. planes) are estimated using the full covariance matrix. The cell esds are taken into account individually in the estimation of esds in distances, angles and torsion angles; correlations between esds in cell parameters are only used when they are defined by crystal symmetry. An approximate (isotropic) treatment of cell esds is used for estimating esds involving l.s. planes.
Refinement. Refinement of *F*^2^ against ALL reflections. The weighted *R*-factor wR and goodness of fit *S* are based on *F*^2^, conventional *R*-factors *R* are based on *F*, with *F* set to zero for negative *F*^2^. The threshold expression of *F*^2^ > σ(*F*^2^) is used only for calculating *R*-factors(gt) etc. and is not relevant to the choice of reflections for refinement. *R*-factors based on *F*^2^ are statistically about twice as large as those based on *F*, and *R*- factors based on ALL data will be even larger.

### Fractional atomic coordinates and isotropic or equivalent isotropic displacement parameters (Å^2^)

**Table:**

	*x*	*y*	*z*	*U*_iso_*/*U*_eq_	
Cl1	0.18059 (15)	0.39628 (9)	0.50346 (4)	0.0390 (2)	
F1	0.7206 (4)	1.0219 (2)	0.35103 (8)	0.0430 (5)	
F2	0.4712 (4)	0.9283 (2)	0.28305 (8)	0.0450 (5)	
F3	0.3535 (5)	1.0974 (2)	0.35501 (11)	0.0629 (7)	
N1	0.8456 (5)	0.7028 (3)	0.30856 (11)	0.0326 (6)	
N2	0.7484 (5)	0.5994 (3)	0.35248 (10)	0.0262 (5)	
N3	0.1542 (5)	0.7067 (3)	0.45907 (11)	0.0308 (6)	
N4	0.4754 (5)	0.5271 (3)	0.42520 (10)	0.0272 (6)	
C1	0.8693 (6)	0.4455 (4)	0.35742 (13)	0.0312 (7)	
H1A	0.8338	0.3531	0.3848	0.037*	
C2	1.0494 (6)	0.4503 (4)	0.31581 (13)	0.0312 (7)	
H2A	1.1661	0.3635	0.3079	0.037*	
C3	1.0253 (6)	0.6127 (4)	0.28693 (13)	0.0332 (7)	
H3A	1.1282	0.6524	0.2552	0.040*	
C4	0.5510 (6)	0.6502 (3)	0.38708 (12)	0.0250 (6)	
C5	0.4336 (6)	0.8113 (4)	0.38345 (13)	0.0283 (6)	
C6	0.2380 (6)	0.8301 (3)	0.42115 (13)	0.0304 (7)	
H6A	0.1561	0.9381	0.4204	0.037*	
C7	0.2831 (6)	0.5640 (3)	0.45766 (12)	0.0296 (7)	
C8	0.4981 (7)	0.9624 (4)	0.34258 (14)	0.0378 (8)	

### Atomic displacement parameters (Å^2^)

**Table:**

	*U*^11^	*U*^22^	*U*^33^	*U*^12^	*U*^13^	*U*^23^
Cl1	0.0460 (5)	0.0364 (3)	0.0347 (4)	−0.0015 (3)	0.0081 (4)	0.0070 (3)
F1	0.0604 (15)	0.0304 (8)	0.0382 (10)	−0.0103 (9)	0.0101 (10)	−0.0007 (7)
F2	0.0489 (13)	0.0564 (12)	0.0298 (10)	0.0079 (10)	−0.0012 (8)	0.0164 (8)
F3	0.0824 (18)	0.0377 (10)	0.0685 (14)	0.0283 (12)	0.0320 (13)	0.0215 (10)
N1	0.0390 (16)	0.0315 (12)	0.0272 (14)	0.0008 (12)	0.0052 (12)	0.0051 (10)
N2	0.0341 (15)	0.0238 (10)	0.0208 (11)	0.0022 (11)	−0.0010 (10)	0.0006 (9)
N3	0.0295 (15)	0.0341 (13)	0.0288 (14)	0.0000 (12)	0.0027 (11)	−0.0013 (10)
N4	0.0355 (16)	0.0256 (10)	0.0205 (12)	−0.0014 (11)	−0.0022 (11)	−0.0010 (9)
C1	0.043 (2)	0.0259 (14)	0.0242 (14)	0.0034 (13)	−0.0011 (14)	−0.0004 (11)
C2	0.0343 (19)	0.0304 (14)	0.0288 (16)	0.0047 (12)	−0.0038 (14)	−0.0053 (12)
C3	0.038 (2)	0.0353 (15)	0.0262 (15)	0.0021 (15)	0.0067 (13)	−0.0006 (13)
C4	0.0296 (16)	0.0269 (13)	0.0185 (13)	−0.0009 (11)	−0.0032 (13)	−0.0021 (11)
C5	0.0361 (18)	0.0272 (14)	0.0215 (14)	0.0029 (12)	−0.0055 (14)	−0.0006 (12)
C6	0.0332 (18)	0.0269 (13)	0.0312 (15)	0.0043 (12)	−0.0014 (14)	−0.0001 (11)
C7	0.038 (2)	0.0311 (15)	0.0198 (13)	−0.0031 (13)	−0.0031 (13)	0.0008 (11)
C8	0.050 (2)	0.0337 (15)	0.0300 (17)	0.0120 (15)	0.0064 (15)	0.0052 (13)

### Geometric parameters (Å, °)

**Table:**

Cl1—C7	1.732 (3)	N4—C4	1.331 (4)
F1—C8	1.336 (4)	C1—C2	1.355 (5)
F2—C8	1.334 (4)	C1—H1A	0.9500
F3—C8	1.345 (4)	C2—C3	1.408 (4)
N1—C3	1.308 (4)	C2—H2A	0.9500
N1—N2	1.360 (3)	C3—H3A	0.9500
N2—C1	1.369 (4)	C4—C5	1.406 (4)
N2—C4	1.392 (4)	C5—C6	1.374 (5)
N3—C7	1.315 (4)	C5—C8	1.511 (4)
N3—C6	1.345 (4)	C6—H6A	0.9500
N4—C7	1.316 (4)		
			
C3—N1—N2	104.4 (2)	N2—C4—C5	125.9 (3)
N1—N2—C1	111.6 (3)	C6—C5—C4	115.4 (3)
N1—N2—C4	122.2 (2)	C6—C5—C8	117.5 (3)
C1—N2—C4	126.2 (2)	C4—C5—C8	127.1 (3)
C7—N3—C6	112.8 (3)	N3—C6—C5	124.7 (3)
C7—N4—C4	116.1 (3)	N3—C6—H6A	117.6
C2—C1—N2	106.8 (3)	C5—C6—H6A	117.6
C2—C1—H1A	126.6	N3—C7—N4	129.7 (3)
N2—C1—H1A	126.6	N3—C7—Cl1	115.5 (2)
C1—C2—C3	104.7 (3)	N4—C7—Cl1	114.7 (2)
C1—C2—H2A	127.6	F2—C8—F1	107.9 (3)
C3—C2—H2A	127.6	F2—C8—F3	106.4 (3)
N1—C3—C2	112.6 (3)	F1—C8—F3	105.3 (3)
N1—C3—H3A	123.7	F2—C8—C5	113.4 (3)
C2—C3—H3A	123.7	F1—C8—C5	113.9 (3)
N4—C4—N2	112.9 (2)	F3—C8—C5	109.6 (3)
N4—C4—C5	121.2 (3)		
